# Supplementary material for: SRBD1 facilitates chromosome segregation by promoting topoisomerase IIα localization to mitotic chromosomes
Source: Nat Commun. 2025 Feb 16;16:1675. doi: 10.1038/s41467-025-56911-w (PMC11830093; doi:10.1038/s41467-025-56911-w)
Supplement: Supplementary file 1 — Supplementary Information [file 41467_2025_56911_MOESM1_ESM.pdf]

**Supplementary Information**

# SRBD1 facilitates chromosome segregation by promoting topoisomerase II$\alpha$ localization to mitotic chromosomes

Courtney A. Lovejoy[1,*], Sarah R. Wessel[1,5], Rahul Bhowmick[1], Yuki Hatoyama[2,3], Masato T. Kanemaki[2,3,4], Runxiang Zhao[1], David Cortez[1,*]

[1]Department of Biochemistry, Vanderbilt University School of Medicine, Nashville, TN 37232, USA

[2]Department of Chromosome Science, National Institute of Genetics, Research Organization of Information and Systems (ROIS), Yata 1111, Mishima, Shizuoka 411-8540, Japan

[3]Graduate School for Advanced Studies, SOKENDAI, Yata 1111, Mishima, Shizuoka, 411-8540, Japan

[4]Department of Biological Science, Graduate School of Science, The University of Tokyo, Tokyo, 113-0033, Japan

[5]Present address: BPGbio, Framingham MA 01701, USA

*Correspondence: david.cortez@vanderbilt.edu and courtney.lovejoy@vanderbilt.edu

**Contents**

Supplementary Figures 1-8

Supplementary References

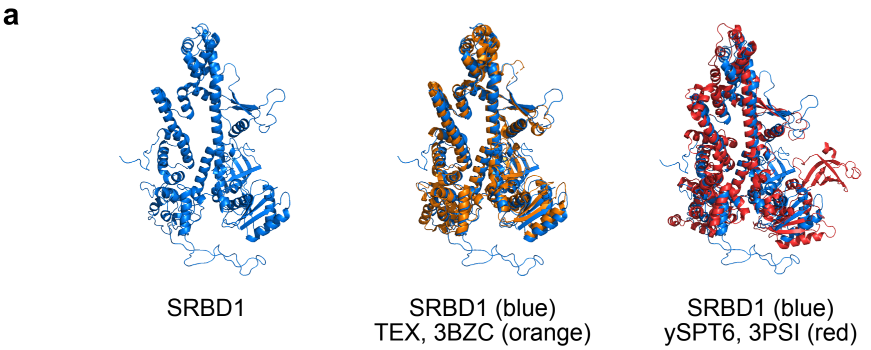


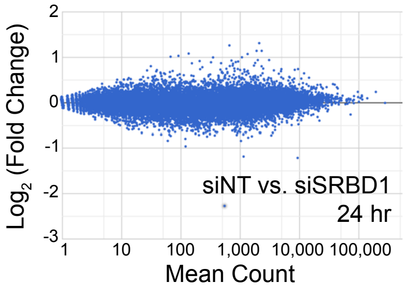


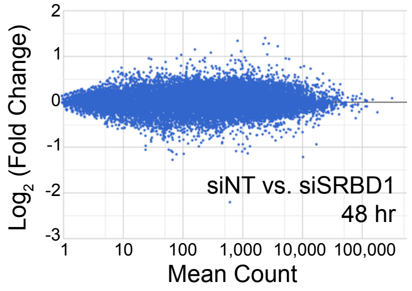


| Gene | $Log_2$ (Fold Change) | q value |
|---|---|---|
| *SRBD1* | -2.27 | 0 |
| *PRR11* | -1.21 | 0 |
| *FKBP14* | -1.18 | 2.09E-30 |
| *HTR2A* | 1.01 | 0.000518 |
| *FAM110D* | 1.03 | 0.0000928 |
| *ZNF367* | 1.07 | 2.96E-34 |
| *ZNF492* | 1.08 | 0.00000536 |
| *GNPNAT1* | 1.14 | 0 |
| *SMIM15* | 1.14 | 2.52E-32 |
| *DESI2* | 1.31 | 1.26E-44 |

| Gene | $Log_2$ (Fold Change) | q value |
|---|---|---|
| *SRBD1* | -2.2 | 0 |
| *NPPB* | -1.27 | 0.000108 |
| *PRR11* | -1.21 | 1.71E-41 |
| *TBX10* | -1.15 | 0.0000149 |
| *ACTBL2* | -1.14 | 0.0000145 |
| *ADM* | -1.13 | 1.48E-09 |
| *TMEM130* | -1.1 | 0.000303 |
| *LEPREL1* | -1.09 | 1.24E-12 |
| *GREM2* | -1.07 | 0.000118 |
| *AGT* | -1.05 | 0.00063 |
| *CBLN2* | -1.05 | 0.00278 |
| *WNT5A* | -1.03 | 4.89E-23 |
| *SBSN* | 1.08 | 0.0000318 |
| *HEATR1* | 1.22 | 0 |
| *SMIM15* | 1.27 | 2.35E-39 |
| *GNPNAT1* | 1.28 | 0 |
| *DESI2* | 1.4 | 0 |

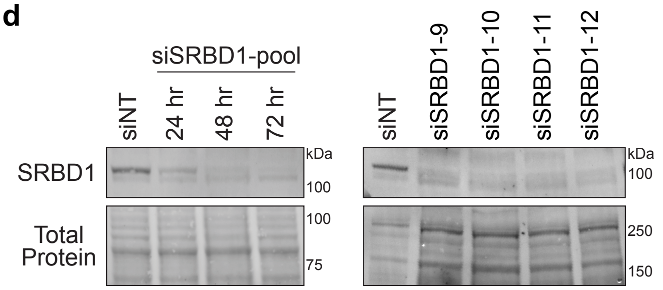


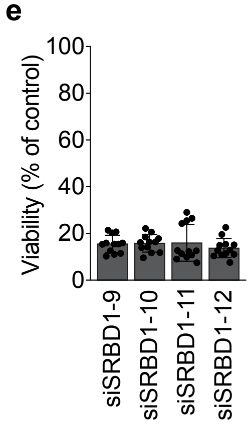


**Supplementary Figure 1. SRBD1 has homology to chromatin remodelers and its inactivation has minimal effects on gene expression.** (a) The predicted AlphaFold structure of SRBD1 (blue, left)

closely resembles the crystal structures of the *P. aeruginosa* Tex (orange, center) and *S. cerevisiae* Spt6 (red, right) proteins**.** (b, c) MA plots for RNA-seq data from U2OS cells comparing non-targeting (NT) and SRBD1 siRNAs at 24 hr (b) and 48 hr (c) after transfection. The $log_2$ fold change for each gene is plotted against the average $log_2$ counts per million. Genes showing significant decreases or increases in expression after siRNA depletion of SRBD1 are colored orange or green, respectively, in the tables below the MA plots. (d) Immunoblots for SRBD1 in U2OS cells transfected with a pool of 4 siRNAs (left), and the knockdown achieved with each individual siRNA at 48 hr after transfection (right), relative to cells transfected with a non-targeting (NT) siRNA. Bio-Rad Stain-Free total protein was used as a loading control. (e) Cell proliferation/viability was measured using alamarBlue after transfection of U2OS cells with the indicated siRNAs. The graph displays the mean +/- SD of the biological replicates (n=12). Source data are provided as a Source Data file.

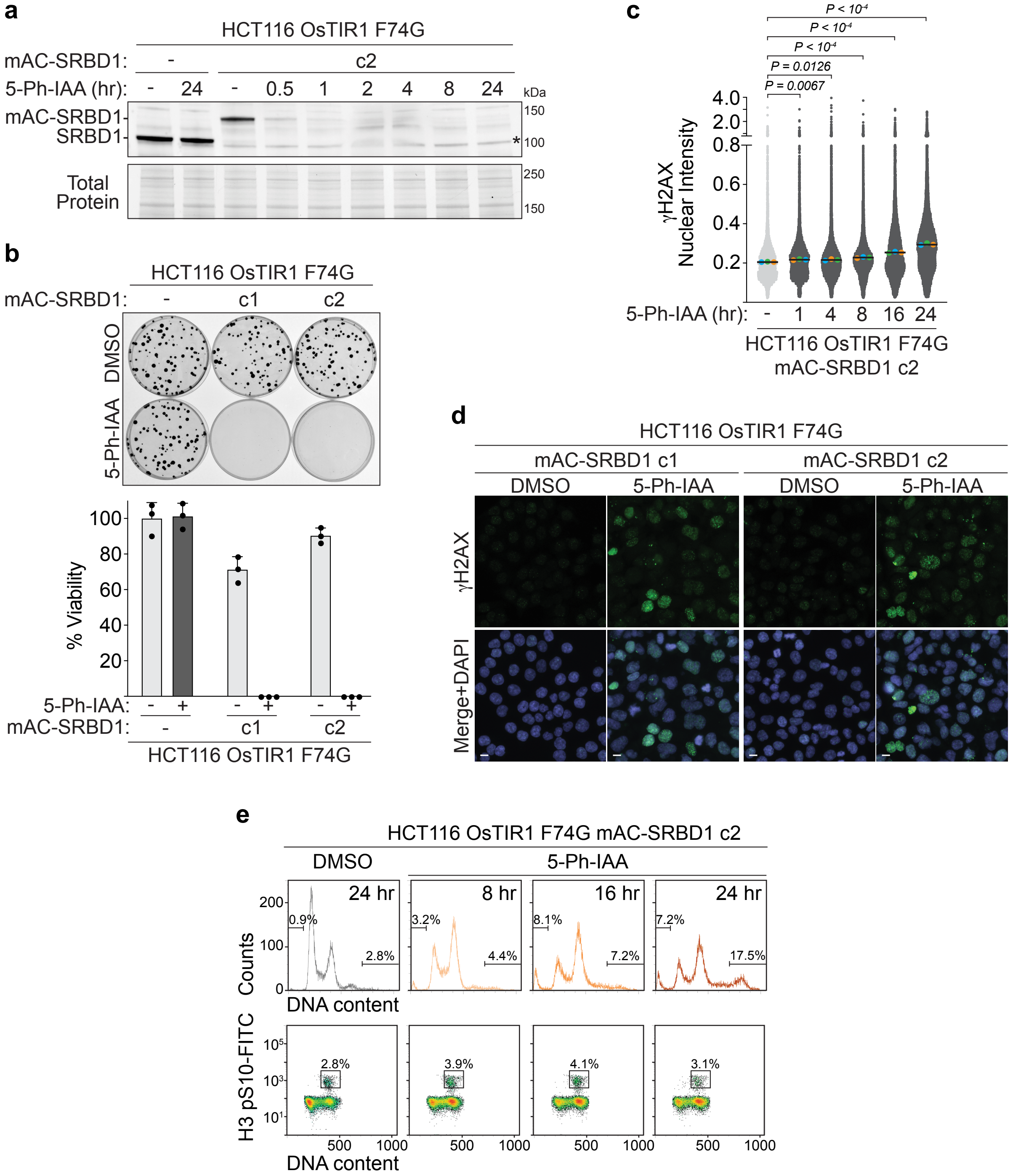


**Supplementary Figure 2. Degradation of SRBD1 causes DNA damage and rapid cell death.** (a) Immunoblot analysis of SRBD1 (parental clone) and mAID2-mClover-SRBD1 (mAC-SRBD1, clone 2) in asynchronous cells treated with DMSO (24 hr) or 1 μM 5-Ph-IAA for the indicated amounts of time. A cross-reacting protein that migrates just below the untagged SRBD1 protein is denoted by an asterisk.

(b) Viability was measured by colony forming ability after a single treatment of DMSO or 1 μM 5-Ph-IAA in parental and degron cells. Representative colony images (top) and quantitation (bottom) are shown. Graph displays the mean +/- SD of the biological replicates (n=3). (c) Asynchronously growing SRBD1 degron cells (c2) were treated with DMSO (24 hr) or 1 μM 5-Ph-IAA for the indicated amounts of time and γH2AX was measured by immunofluorescence imaging. Each gray data point represents the nuclear intensity in one cell (arbitrary units x $10^7$; total cells analyzed ≥10,258). The colored data points represent the mean of each biological replicate (n=3) and black bars represent the mean of the three replicates. Significance was determined using a one-way ANOVA with Dunnett's multiple comparisons test comparing the means of replicate experiments. (d) Representative images of γH2AX immunostaining (green) in asynchronously growing SRBD1 degron cells (c1 and c2) treated with DMSO or 1 μM 5-Ph-IAA (24 hr). DNA is stained with DAPI (blue). Scale bars represent 10 μm. (e) Asynchronously growing SRBD1 degron cells (c2) were treated with DMSO (24 hr) or 1 μM 5-Ph-IAA for the indicated amounts of time. Cell cycle distributions (from 25,000 gated cells) were analyzed by flow cytometry and pH3 staining was used to differentiate mitotic and G2 phase cells. Source data are provided as a Source Data file.

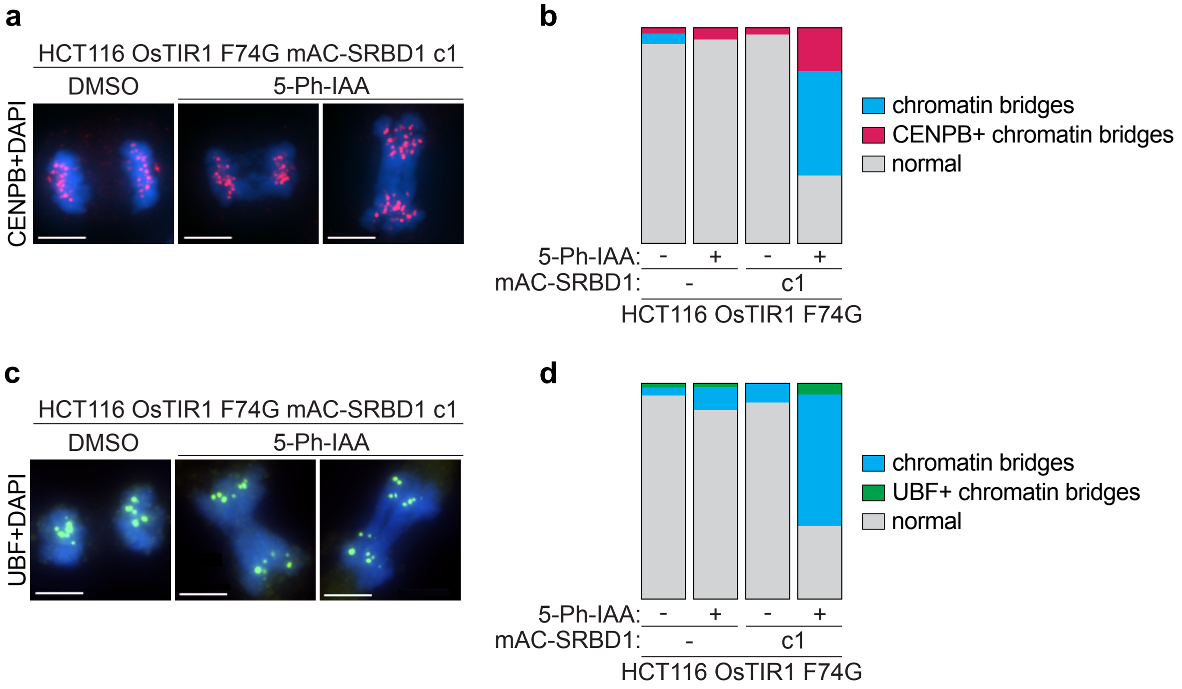


**Supplementary Figure 3. The anaphase chromatin bridges observed after SRBD1 degradation are not derived from a particular locus.** G2 phase synchronized cells were treated with DMSO or 1 μM 5-Ph-IAA for 1 hr prior to release into mitosis. Centromeric and ribosomal DNA were identified in mitotic cells to assess the contribution of these loci to the anaphase chromatin bridges observed after SRBD1 degradation. Centromeres were visualized by CENPB immunostaining (a) and chromatin bridges with CENPB foci (red) at the bridge termini were quantified (b). Ribosomal DNA was visualized by immunostaining UBF (c) and chromatin bridges with UBF foci (green) at the bridge termini were quantified (d). DNA is stained with DAPI (blue). All scale bars represent 5 μm. CENPB staining is representative of a single experiment (total cells analyzed ≥32) and UBF staining is representative of two biological replicates (total cells analyzed ≥52), with similar results observed in the second degron clone. Bars represent the fraction of anaphases displaying the indicated phenotypes (b, d). Source data are provided as a Source Data file.

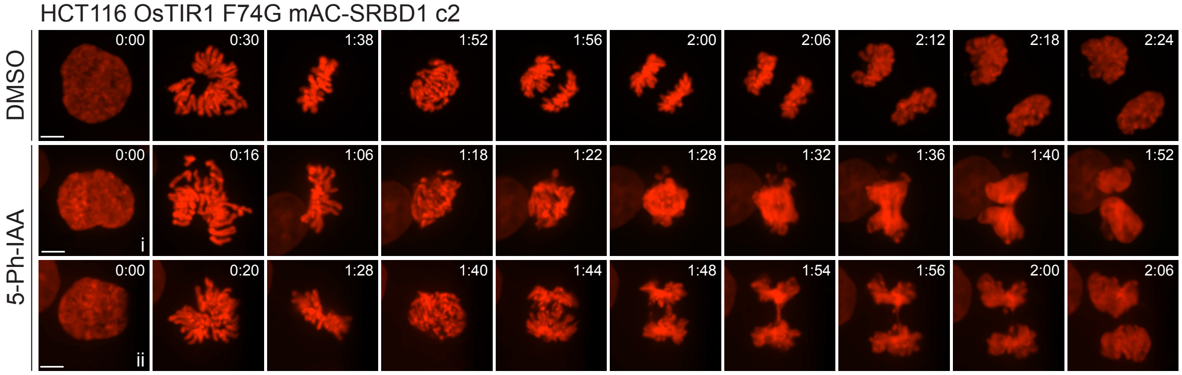


**Supplementary Figure 4. SRBD1 inactivation results in mitotic failure.** Still pictures derived from live imaging of histone H2B-mCherry in SRBD1 degron clone 2. G2 phase synchronized cells were treated with DMSO or 1 μM 5-Ph-IAA for 1 hr and released into mitosis. A comparable time point in prophase is shown for each, with subsequent panels displaying prometaphase, metaphase, the initiation of anaphase, and comparable time points thereafter. A normal anaphase in DMSO-treated cells is shown, along with representative images of the anaphase failure (i) and chromatin bridges (ii) observed after 5-Ph-IAA-induced degradation of SRBD1. Scale bars represent 5 μm.

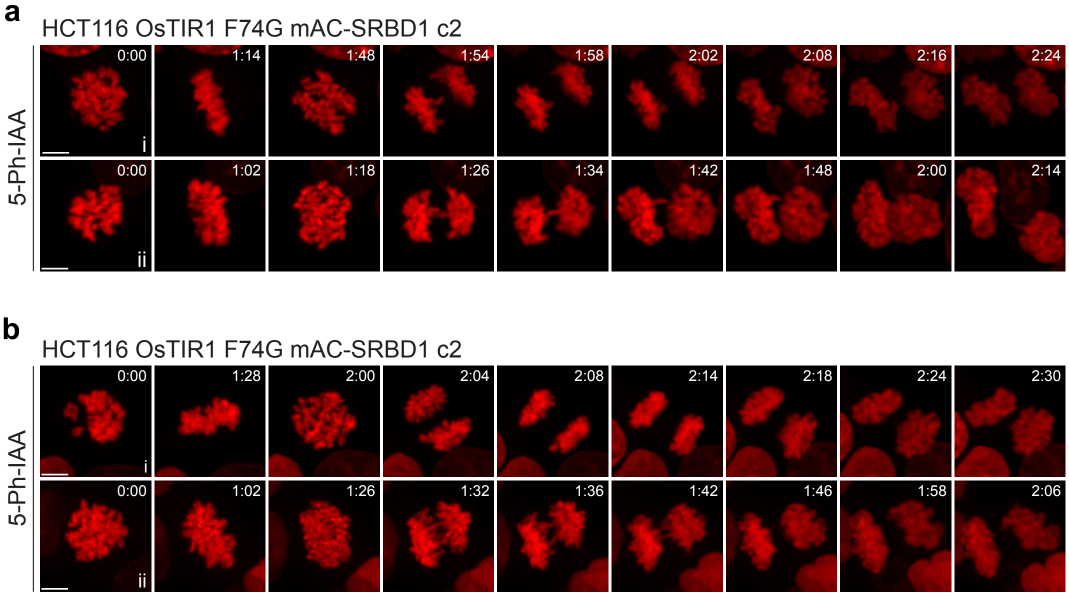


**Supplementary Figure 5. SRBD1 function is critical in early mitosis to prevent anaphase failure.** Still pictures derived from live imaging of histone H2B-mCherry in SRBD1 degron clone 2. (a) Anaphase defects were visualized after degradation of SRBD1 during prometaphase. The first panel displays the nocodazole-synchronized cells at the start of imaging, with subsequent panels showing metaphase, the initiation of anaphase, and comparable time points thereafter. Normal anaphase progression (i) and chromatin bridges (ii) were observed after 5-Ph-IAA-induced degradation of SRBD1 during prometaphase. (b) SRBD1 was degraded in G2 phase synchronized cells, mitotic progression was delayed for 2 hr in prometaphase with nocodazole, and anaphase progression was visualized upon release. The first panel displays the prometaphase-synchronized cells at the start of imaging, with subsequent panels showing metaphase, the initiation of anaphase, and comparable time points after. Normal anaphase progression (i) and chromatin bridges (ii) were observed after 5-Ph-IAA-induced degradation of SRBD1. All scale bars represent 5 μm.

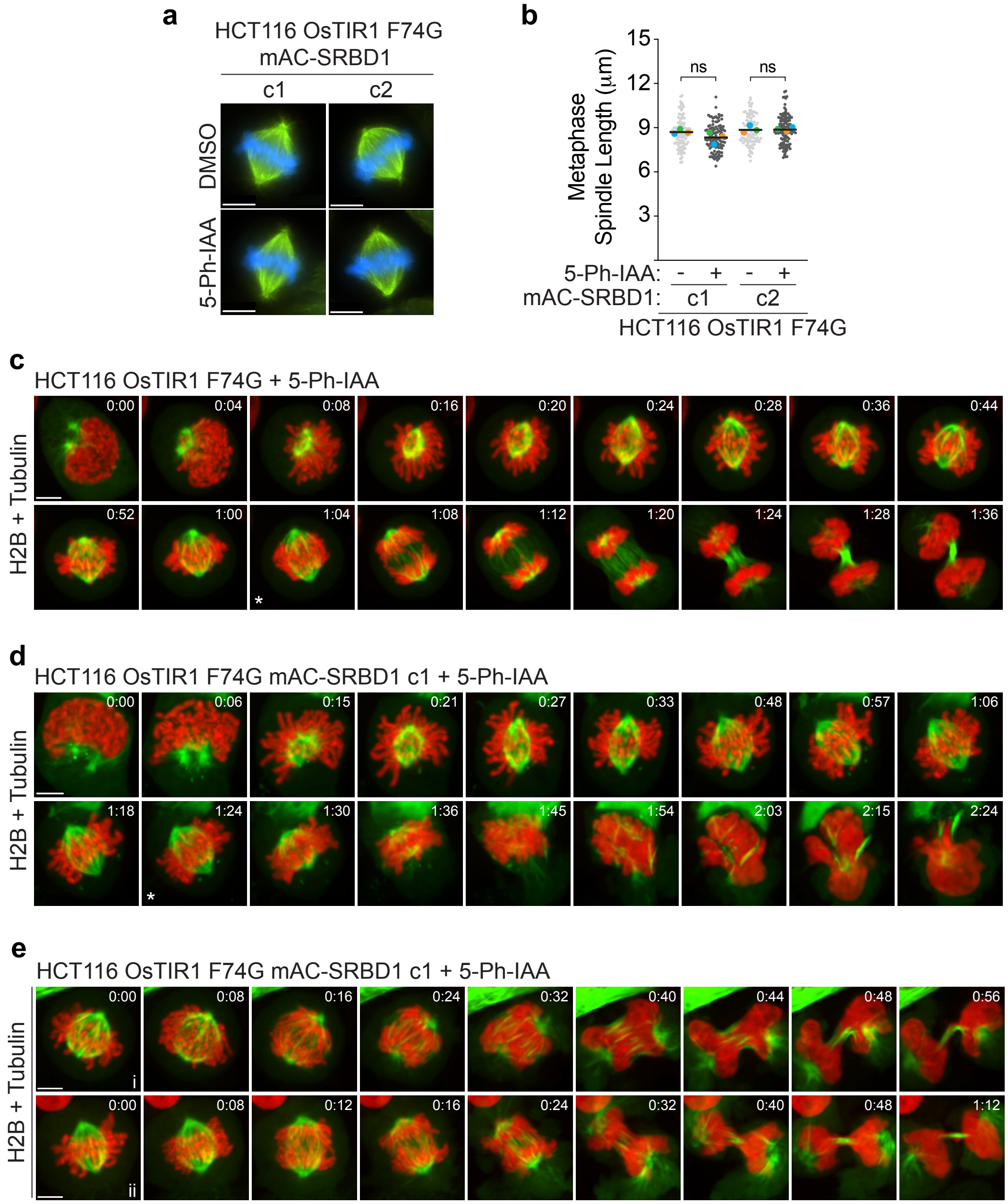


**Supplementary Figure 6. The mitotic spindle appears normal until anaphase initiation in SRBD1-deficient cells.** (a, b) G2 phase synchronized cells were treated with DMSO or 1 μM 5-Ph-IAA for 1 hr and mitotic spindles were visualized by immunostaining. (a) Representative images of αtubulin staining (green) on metaphase cells. DNA is stained with DAPI (blue). (b) Quantitation of the pole-to-pole spindle length of metaphase cells. Each gray data point represents the spindle length in one metaphase cell

(total cells analyzed ≥105). Colored data points represent the mean of each biological replicate (n=3). Black bars represent the mean of the three replicates and significance was determined using a two-sided, paired t-test comparing the means of replicate experiments. (c-e) Still pictures derived from live imaging of cells expressing histone H2B-mCherry (red) and tubulin-GFP (green). Asynchronously growing cells were treated with 1 μM 5-Ph-IAA for 1 hr and cells entering mitosis within the next hour were analyzed. The formation and function of the mitotic spindle is shown in a control cell from prophase through telophase (c), and after G2 phase degradation of SRBD1 from prophase through a failed mitosis and the subsequent chromosome decondensation (d). The panel displaying anaphase initiation is marked for each with an asterisk. (e) Additional examples (i, ii) of mitotic spindle dynamics in SRBD1-deficient cells with chromosome segregation errors. The first and second panels display metaphase and the initiation of anaphase, respectively, with subsequent panels showing relevant or comparable time points thereafter. All scale bars represent 5 μm. Source data are provided as a Source Data file.

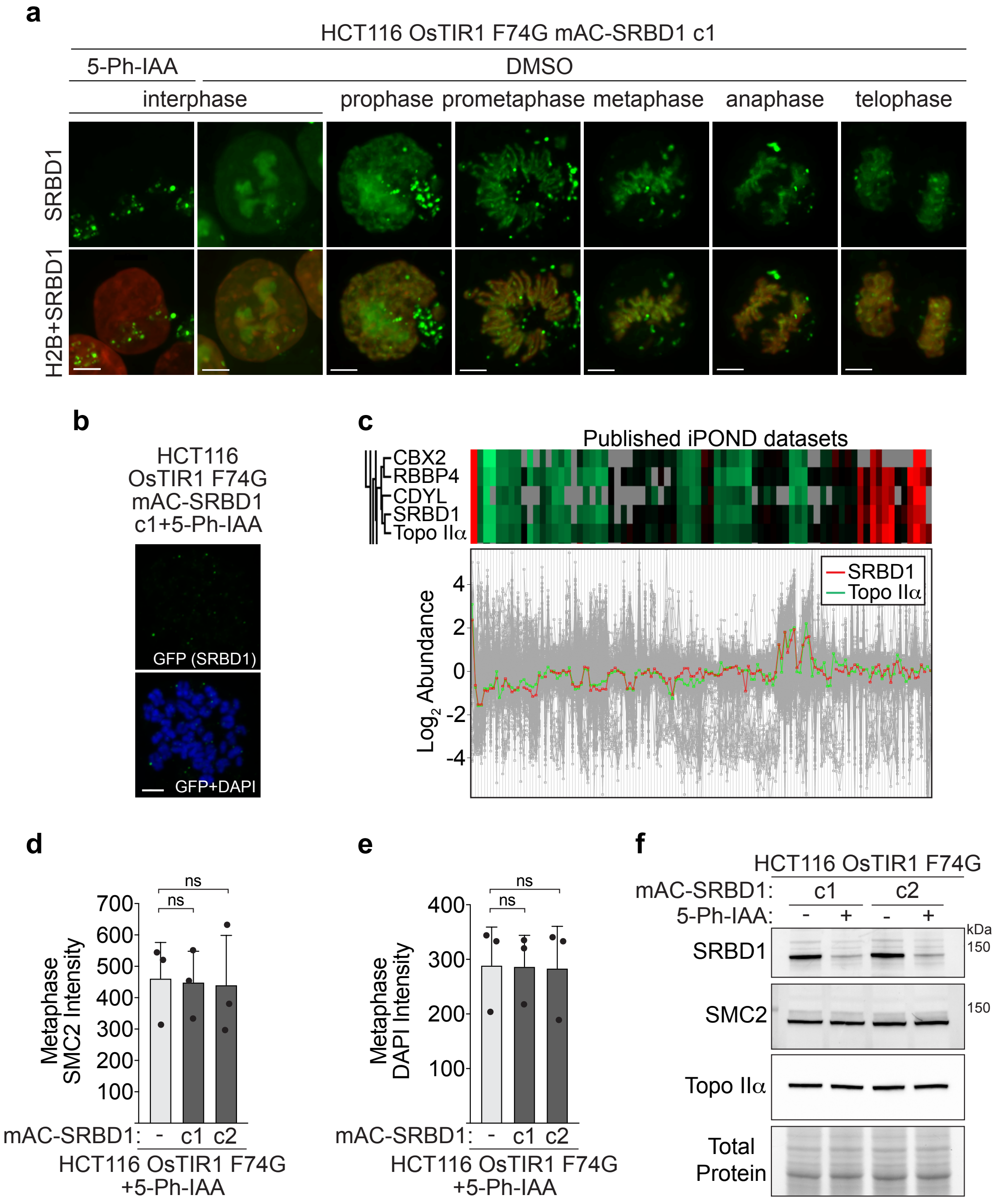


**Supplementary Figure 7. Changes in mitotic topo IIα localization are not attributable to defects in SMC2 localization or aberrant degradation of mitotic scaffold proteins.** (a) Still images derived from live imaging of histone H2B-mCherry (red) and SRBD1 (mClover, green). The nuclear and nucleolar localization of SRBD1 is lost following treatment with 1 μM 5-Ph-IAA for 1 hr in G2 phase synchronized cells (first two panels). Subsequent panels show SRBD1 localization at different mitotic

stages. Note that the very bright spots in the images are not SRBD1 since they remain visible after degradation of SRBD1 with 5-Ph-IAA. Scale bars represent 5 μm. (b) Specificity of SRBD1 immunostaining (GFP, green) on metaphase chromosomes shown by the absence of signal after 5-Ph-IAA-induced degradation. DAPI is in blue. Scale bar represents 5 μm. (c) Hierarchical clustering of iPOND proteomics data[1,2] shows that the abundance of SRBD1 and topo IIα on nascent DNA are correlated. The heat map displays the relative abundance of the indicated proteins across all experiments (green, decreased; red, increased; black, unchanged; gray, not detected). The line graph shows the same data for SRBD1 and topo IIα, with the abundance of all proteins in each experiment shown for comparison in gray. (d, e) G2 phase synchronized cells were treated with 1 μM 5-Ph-IAA for 1 hr and mitotic cells were collected for immunostaining of metaphase chromosome spreads. The intensity of SMC2 (d) and DAPI (e) on metaphase chromosomes was quantified using CellProfiler (arbitrary units; total chromosomes analyzed ≥1,879). Graphs display the mean chromosomal intensity of the biological replicates +/- SD (n=3). Significance was determined using a one-way ANOVA with Dunnett's multiple comparisons test comparing the means of the replicate experiments. (f) G2 phase synchronized cells were treated with DMSO or 1 μM 5-Ph-IAA for 1 hr, released into nocodazole for 45 min, and mitotic cells were collected by shake-off. SRBD1 and the mitotic scaffold proteins SMC2 and topo IIα were analyzed by immunoblotting. Bio-Rad Stain-Free total protein was used as a loading control. Source data are provided as a Source Data file.

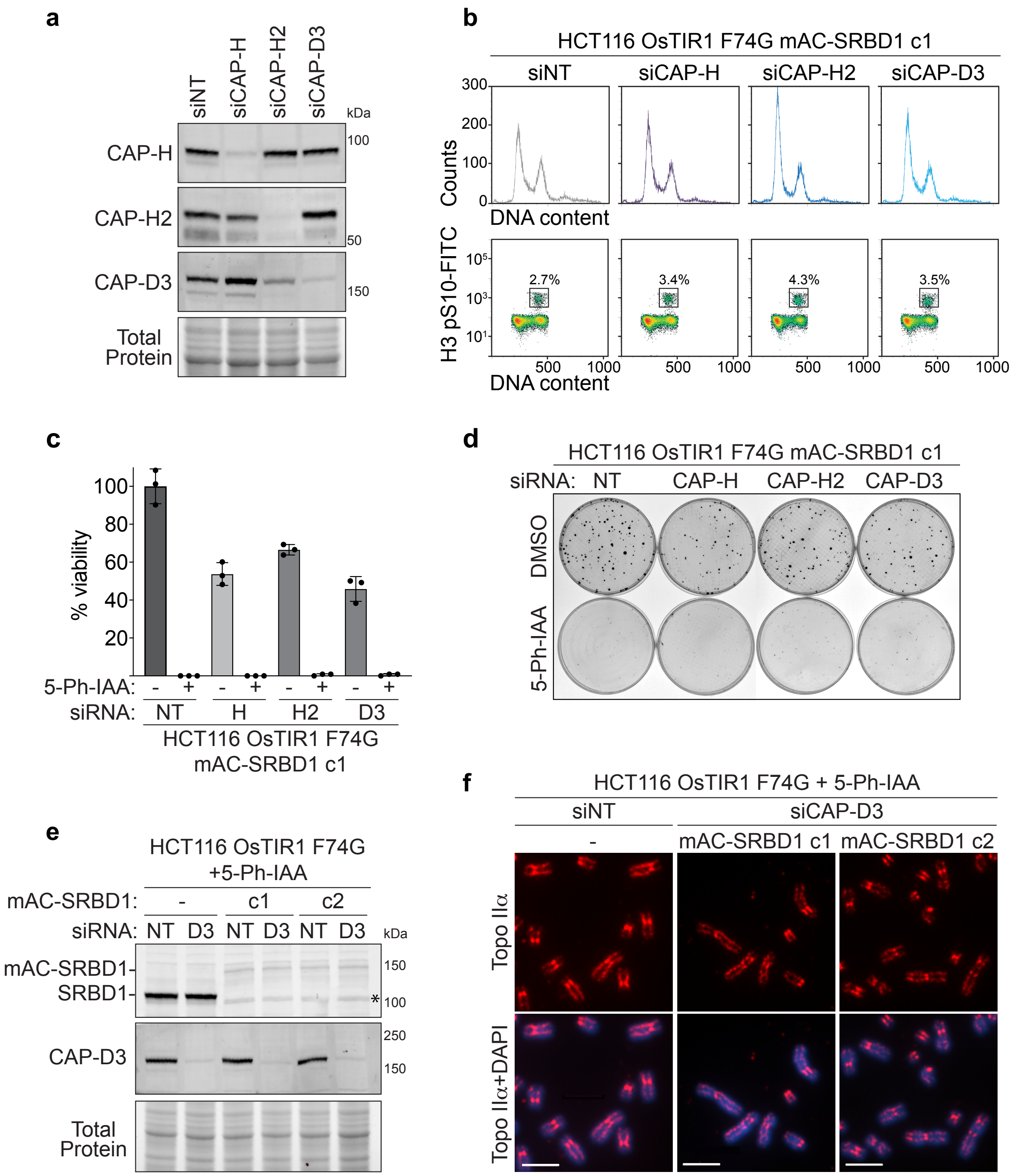


**Supplementary Figure 8. Efficient knockdown of Condensin I and II subunits by siRNA transfection does not dramatically alter cell proliferation or mitotic progression.** (a) Immunoblot showing depletion of the indicated condensin subunits 48 hr after siRNA transfection. Bio-Rad Stain-Free total protein was used as a loading control. (b) Asynchronously growing cells were collected 48 hr after siRNA transfection and cell cycle distributions were analyzed by flow cytometry on 25,000 gated

cells. pH3 staining was used to differentiate mitotic and G2 phase cells. (c, d) Viability was measured by colony forming ability after a single treatment of DMSO or 1 μM 5-Ph-IAA at 48 hr after transfection with the indicated siRNAs. Quantitation (c) and representative colony images (d) are shown. Graph displays the mean +/- SD of technical replicates (n=3) and is representative of a single experiment. (e) Cells transfected with non-targeting or CAP-D3 siRNAs were synchronized in G2 phase and treated with 1 μM 5-Ph-IAA for 1 hr. Cells were released into demecolcine for 35-45 min and mitotic cells were collected for immunoblot analysis of SRBD1 (parental clone), mAID2-mClover-SRBD1 (mAC-SRBD1, clones 1 and 2), and CAP-D3. A cross-reacting protein that migrates just below the untagged SRBD1 protein is denoted by an asterisk. (f) Enlarged images of topo IIα staining on select metaphase chromosome spreads, with Fig. 7e. Scale bars represent 5 μm. Source data are provided as a Source Data file.

## Supplementary References

1. Wessel, S. R., Mohni, K. N., Luzwick, J. W., Dungrawala, H. & Cortez, D. Functional Analysis of the Replication Fork Proteome Identifies BET Proteins as PCNA Regulators. *Cell Rep* **28**, 3497–3509.e4 (2019).

2. Dungrawala, H. *et al.* The Replication Checkpoint Prevents Two Types of Fork Collapse without Regulating Replisome Stability. *Mol Cell* **59**, 998–1010 (2015).
