# Supplementary material for: SRBD1 facilitates chromosome segregation by promoting topoisomerase IIα localization to mitotic chromosomes
Source: Nat Commun. 2025 Feb 16;16:1675. doi: 10.1038/s41467-025-56911-w (PMC11830093; doi:10.1038/s41467-025-56911-w)
Supplement: Supplementary file 2 — Description of Additional Supplementary Files [file 41467_2025_56911_MOESM2_ESM.pdf]

## Description of Additional Supplementary Files

**Supplementary Movie 1.** Normal anaphase progression in SRBD1-proficient cells; related to Fig. 4. Representative movie of H2B-mCherry-labelled degron clone 1, synchronized in G2 phase for treatment with DMSO and released into mitosis.

**Supplementary Movie 2.** Normal anaphase progression in SRBD1-proficient cells; related to Supplementary Fig. 4. Representative movie of H2B-mCherry-labelled degron clone 2, synchronized in G2 phase for treatment with DMSO and released into mitosis.

**Supplementary Movie 3.** Anaphase failure after SRBD1 degradation; related to Fig. 4. Representative movie of H2B-mCherry-labelled degron clone 1, synchronized in G2 phase for treatment with 1 μM 5-Ph-IAA (1 hr) and released into mitosis.

**Supplementary Movie 4.** Anaphase failure after SRBD1 degradation; related to Supplementary Fig. 4. Representative movie of H2B-mCherry-labelled degron clone 2, synchronized in G2 phase for treatment with 1 μM 5-Ph-IAA (1 hr) and released into mitosis.

**Supplementary Movie 5.** Mitotic spindle dynamics in a control cell; related to Supplementary Fig. 6c. Representative movie of mitotic progression in asynchronously growing control cells expressing H2B-mCherry and tubulin-GFP after treatment with 1 μM 5-Ph-IAA (1 hr).

**Supplementary Movie 6.** Mitotic spindle dynamics after G2 phase inactivation of SRBD1; related to Supplementary Fig. 6d. Representative movie of anaphase failure in asynchronously growing degron clone 1 expressing H2B-mCherry and tubulin-GFP after treatment with 1 μM 5-Ph-IAA (1 hr).

**Supplementary Movie 7.** Normal anaphase progression in SRBD1-proficient cells after transfection with non-targeting siRNA; related to Fig. 7. Representative movie of H2B-mCherry-labelled degron clone 1, synchronized in G2 phase for treatment with DMSO and released into mitosis 48 hr after siRNA transfection.

**Supplementary Movie 8.** Anaphase failure after SRBD1 degradation in cells transfected with non-targeting siRNA; related to Fig. 7. Representative movie of H2B-mCherry-labelled degron clone 1, synchronized in G2 phase for treatment with 1 μM 5-Ph-IAA (1 hr) and released into mitosis 48 hr after siRNA transfection.

**Supplementary Movie 9.** Anaphase failure after SRBD1 degradation in cells transfected with CAP-H siRNA; related to Fig. 7. Representative movie of H2B-mCherry-labelled degron clone 1, synchronized in G2 phase for treatment with 1 μM 5-Ph-IAA (1 hr) and released into mitosis 48 hr after siRNA transfection.

**Supplementary Movie 10.** Anaphase chromatin bridging after SRBD1 degradation in cells transfected with CAP-H2 siRNA; related to Fig. 7. Representative movie of H2B-mCherry-labelled degron clone 1, synchronized in G2 phase for treatment with 1 μM 5-Ph-IAA (1 hr) and released into mitosis 48 hr after siRNA transfection.

**Supplementary Movie 11.** Normal anaphase progression after SRBD1 degradation in cells transfected with CAP-D3 siRNA; related to Fig. 7. Representative movie of H2B-mCherry-labelled degron clone 1, synchronized in G2 phase for treatment with 1 μM 5-Ph-IAA (1 hr) and released into mitosis 48 hr after siRNA transfection.
