# Supplementary material for: SRBD1 facilitates chromosome segregation by promoting topoisomerase IIα localization to mitotic chromosomes
Source: Nat Commun. 2025 Feb 16;16:1675. doi: 10.1038/s41467-025-56911-w (PMC11830093; doi:10.1038/s41467-025-56911-w)
Supplement: Supplementary file 14 — Reporting Summary [file 41467_2025_56911_MOESM14_ESM.pdf]

nature portfolio

Corresponding author(s): David Cortez and Courtney Lovejoy

Last updated by author(s): Dec 17, 2024

Reporting Summary

Nature Portfolio wishes to improve the reproducibility of the work that we publish. This form provides structure for consistency and transparency in reporting. For further information on Nature Portfolio policies, see our Editorial Policies and the Editorial Policy Checklist.

Statistics

For all statistical analyses, confirm that the following items are present in the figure legend, table legend, main text, or Methods section.

n/a	Confirmed	
☐	☒	The exact sample size (*n*) for each experimental group/condition, given as a discrete number and unit of measurement
☒	☐	A statement on whether measurements were taken from distinct samples or whether the same sample was measured repeatedly
☐	☒	The statistical test(s) used AND whether they are one- or two-sided<br>*Only common tests should be described solely by name; describe more complex techniques in the Methods section.*
☒	☐	A description of all covariates tested
☒	☐	A description of any assumptions or corrections, such as tests of normality and adjustment for multiple comparisons
☐	☒	A full description of the statistical parameters including central tendency (e.g. means) or other basic estimates (e.g. regression coefficient) AND variation (e.g. standard deviation) or associated estimates of uncertainty (e.g. confidence intervals)
☒	☐	For null hypothesis testing, the test statistic (e.g. *F*, *t*, *r*) with confidence intervals, effect sizes, degrees of freedom and *P* value noted<br>*Give P values as exact values whenever suitable.*
☒	☐	For Bayesian analysis, information on the choice of priors and Markov chain Monte Carlo settings
☒	☐	For hierarchical and complex designs, identification of the appropriate level for tests and full reporting of outcomes
☒	☐	Estimates of effect sizes (e.g. Cohen's *d*, Pearson's *r*), indicating how they were calculated

Our web collection on statistics for biologists contains articles on many of the points above.

Software and code

Policy information about availability of computer code

Data collection	Immunofluorescence data was collected on a Molecular Devices ImageXpress. Static anaphase or metaphase images were captured on a Nikon Eclipse Ti. Live cell images were collected on a Yokogawa CSU-X1 spinning disk field scanning confocal system.
Data analysis	Molecular Devices MetaXpress; Nikon Elements; Cell Profiler; Prism 10

For manuscripts utilizing custom algorithms or software that are central to the research but not yet described in published literature, software must be made available to editors and reviewers. We strongly encourage code deposition in a community repository (e.g. GitHub). See the Nature Portfolio guidelines for submitting code & software for further information.

Data

Policy information about availability of data

All manuscripts must include a data availability statement. This statement should provide the following information, where applicable:

- Accession codes, unique identifiers, or web links for publicly available datasets
- A description of any restrictions on data availability
- For clinical datasets or third party data, please ensure that the statement adheres to our policy

The RNA-seq data is available in Geo repository under accession number GSE283162. The proteomics data is deposited in PRIDE under accession number PXD020914. All other data is provided in the manuscript, the supplementary information, and the source data files.

Research involving human participants, their data, or biological material

Policy information about studies with human participants or human data. See also policy information about sex, gender (identity/presentation), and sexual orientation and race, ethnicity and racism.

Reporting on sex and gender	not applicable
Reporting on race, ethnicity, or other socially relevant groupings	not applicable
Population characteristics	not applicable
Recruitment	not applicable
Ethics oversight	not applicable

Note that full information on the approval of the study protocol must also be provided in the manuscript.

Field-specific reporting

Please select the one below that is the best fit for your research. If you are not sure, read the appropriate sections before making your selection.

- [x] Life sciences
- [ ] Behavioural & social sciences
- [ ] Ecological, evolutionary & environmental sciences

For a reference copy of the document with all sections, see nature.com/documents/nr-reporting-summary-flat.pdf

Life sciences study design

All studies must disclose on these points even when the disclosure is negative.

Sample size	No sample size calculation was performed. Numbers of cells analyzed were based on how many could be imaged within an experiment.
Data exclusions	No data exclusions.
Replication	Numbers of replicate experiments are indicated in the figure legends.
Randomization	Not applicable.
Blinding	Investigator was blinded to sample identities during most analyses. The exception was the live cell imaging, for which samples could only be imaged one at a time.

Reporting for specific materials, systems and methods

We require information from authors about some types of materials, experimental systems and methods used in many studies. Here, indicate whether each material, system or method listed is relevant to your study. If you are not sure if a list item applies to your research, read the appropriate section before selecting a response.

Materials & experimental systems

n/a	Involved in the study	
☐	☒	Antibodies
☐	☒	Eukaryotic cell lines
☒	☐	Palaeontology and archaeology
☒	☐	Animals and other organisms
☒	☐	Clinical data
☒	☐	Dual use research of concern
☒	☐	Plants

Methods

n/a	Involved in the study	
☒	☐	ChIP-seq
☐	☒	Flow cytometry
☒	☐	MRI-based neuroimaging

Antibodies

Antibodies used	SRBD1 (Bethyl, A305-121A); GAPDH (MilliporeSigma, MAB374); histone H2A (abcam, ab18255); histone H2B (abcam, ab1790); histone H3 (abcam, ab10799); histone H4 (abcam, ab31830); TOP2A (Santa Cruz Biotechnology, sc-166934); SMC2 (abcam, ab10412 or W. Earnshaw); CAP-H (Bethyl, A300-603A); CAP-H2 (abcam, ab200659); CAP-D3 (Bethyl, ab200659); gH2AX (MilliporeSigma, 05-636); pS4,S8-RPA (Bethyl, A300-245A); RPA32 (abcam, ab2175); MoBU-1 (ThermoFisher Scientific, B35128); PICH (Millipore, 04-1540); BLM (abcam, ab2179); CENPB (abcam, ab25734); UBF (Santa Cruz Biotechnology, sc-13125); GFP (Proteintech, pabg1); tubulin (DM1A; Millipore Sigma, T9026); pS10 histone H3 (Cell Signaling Technology, 3377).
Validation	SRBD1, GFP, TOP2A, SMC2, CAP-H, CAP-H2, CAP-D3, PICH, BLM, and tubulin antibodies were validated based on established localization patterns or knocking down target proteins. All other antibodies were validated by the manufacturer or previous publications.

Eukaryotic cell lines

Policy information about cell lines and Sex and Gender in Research

Cell line source(s)	U2OS (ATCC, HTB-96; osteosarcoma, human female origin), HEK293T cells (ATCC, CRL-3216; epithelial, human female origin), HCT116 cells (ATCC, CCL-247; carcinoma, human male origin)
Authentication	Cell lines were authenticated by PCR and immunoblotting.
Mycoplasma contamination	Cell lines were tested using the Universal Mycoplasma Detection kit; ATCC
Commonly misidentified lines (See ICLAC register)	not applicable

Plants

Seed stocks	not applicable
Novel plant genotypes	not applicable
Authentication	not applicable

Flow Cytometry

Plots

Confirm that:

- [x] The axis labels state the marker and fluorochrome used (e.g. CD4-FITC).
- [x] The axis scales are clearly visible. Include numbers along axes only for bottom left plot of group (a 'group' is an analysis of identical markers).
- [x] All plots are contour plots with outliers or pseudocolor plots.
- [x] A numerical value for number of cells or percentage (with statistics) is provided.

Methodology

Sample preparation	Cells were fixed with ice cold 70% ethanol, permeabilized with 0.25% Triton X-100 on ice, and treated with propidium iodide and RNase A. Where indicated, cells were additionally stained with pS10 histone H3 antibody (Cell Signaling Technology, 3377).
Instrument	Attune NxT flow cytometer
Software	Attune Cytometric software
Cell population abundance	*Describe the abundance of the relevant cell populations within post-sort fractions, providing details on the purity of the samples and how it was determined.*

Gating strategy	Cells were gated based on side vs. forward scatter

☒ Tick this box to confirm that a figure exemplifying the gating strategy is provided in the Supplementary Information.
